# Optimization of preparation conditions for *Salsola laricifolia* protoplasts using response surface methodology and artificial neural network modeling

**DOI:** 10.1186/s13007-024-01180-9

**Published:** 2024-04-07

**Authors:** Hao Guo, Yuxin Xi, Kuerban Guzailinuer, Zhibin Wen

**Affiliations:** 1 State Key Laboratory of Desert and Oasis Ecology, Key Laboratory of Ecological Safety and Sustainable Development in Arid Lands, Xinjiang Institute of Ecology and Geography, Chinese Academy of Sciences, Urumqi, 830011 China; 2 College of Life Sciences, Shihezi University, Shihezi, 832003 China; 3 Xinjiang Production and Construction Corps Key Laboratory of Oasis Town and Mountain-Basin System Ecology, Shihezi, 832003 China; 4 Xinjiang Key Lab of Conservation and Utilization of Plant Gene Resources, Urumqi, 830011 China; 5 University of Chinese Academy of Sciences, Beijing, 100049 China; 6 Sino-Tajikistan Joint Laboratory for Conservation and Utilization of Biological Resources, Urumqi, 830011 China; 7 The Specimen Museum of Xinjiang Institute of Ecology and Geography, Chinese Academy of Sciences, Urumqi, 830011 China

**Keywords:** *Salsola laricifolia*, Protoplasts, Response surface methodology, Artificial neural network, Number of viable cells

## Abstract

**Background:**

*Salsola laricifolia* is a typical C_3_–C_4_ typical desert plant, belonging to the family Amaranthaceae*.* An efficient single-cell system is crucial to study the gene function of this plant. In this study, we optimized the experimental conditions by using Box-Behnken experimental design and Response Surface Methodology (RSM)-Artificial Neural Network (ANN) model based on the previous studies.

**Results:**

Among the 17 experiment groups designed by Box-Behnken experimental design, the maximum yield (1.566 × 10^6^/100 mg) and the maximum number of viable cells (1.367 × 10^6^/100 mg) were obtained in group 12, and the maximum viability (90.81%) was obtained in group 5. Based on these results, both the RSM and ANN models were employed for evaluating the impact of experimental factors. By RSM model, cellulase R-10 content was the most influential factor on protoplast yield, followed by macerozyme R-10 content and mannitol concentration. For protoplast viability, the macerozyme R-10 content had the highest influence, followed by cellulase R-10 content and mannitol concentration. The RSM model performed better than the ANN model in predicting yield and viability. However, the ANN model showed significant improvement in predicting the number of viable cells. After comprehensive evaluation of the protoplast yield, the viability and number of viable cells, the optimal results was predicted by ANN yield model and tested. The amount of protoplast yield was 1.550 × 10^6^/100 mg, with viability of 90.65% and the number of viable cells of 1.405 × 10^6^/100 mg. The corresponding conditions were 1.98% cellulase R-10, 1.00% macerozyme R-10, and 0.50 mol L^−1^ mannitol. Using the obtained protoplasts, the reference genes (*18SrRNA*, *β-actin* and *EF1-α*) were screened for expression, and transformed with PEG-mediated *pBI121-SaNADP-ME2-GFP* plasmid vector. There was no significant difference in the expression of *β-actin* and *EF1-α* before and after treatment, suggesting that they can be used as internal reference genes in protoplast experiments. And *SaNADP-ME2* localized in chloroplasts.

**Conclusion:**

The current study validated and evaluated the effectiveness and results of RSM and ANN in optimizing the conditions for protoplast preparation using *S. laricifolia* as materials. These two methods can be used independently of experimental materials, making them suitable for isolating protoplasts from other plant materials. The selection of the number of viable cells as an evaluation index for protoplast experiments is based on its ability to consider both protoplast yield and viability. The findings of this study provide an efficient single-cell system for future genetic experiments in *S. laricifolia* and can serve as a reference method for preparing protoplasts from other materials.

**Supplementary Information:**

The online version contains supplementary material available at 10.1186/s13007-024-01180-9.

## Background

Protoplast is a general term for a variety of substances in plant cells other than the cell wall removed, which has the totipotency as well as a part of the viability of ordinary plant cells [1], and have been extensively utilized in various experiments, such as gene promoter screening, verification of exogenous gene function, subcellular localization of proteins, protein interactions and multiplex genome editing [2–5] Protoplast systems of model plants, such as *Arabidopsis thaliana* and *Nicotiana tabacum*, have been extensively used in gene function studies. However, it is important to note that due to the genetic variations among different plant protoplasts, these systems may provide inaccurate expression information for exogenous genes [6]. Therefore, in recent years, many non-model plants have also developed their own protoplast preparation systems to verify and explore gene functions. Some examples include *Apium graveolens*, *Camellia sinensis*, *Gossypium spp*, *Panicum virgatum*, *Populus tomentosa* [7–12].

Protoplast preparation was influenced by plant materials, sampling locations, and growth time [13, 14]. The amount of protoplast yield in *Oryza sativa* callus was 2.500 × 10^6^/g FW through a 3 h enzymatic digestion. The digestion process involved the use of 1.50% cellulase, 1.00% macerozyme, and 0.60 mol L^−1^ mannitol [3]. In the case of *Colobanthus quitensis* leaves, enzymatic digestion for 3 h was carried out. The digestion solution contained 3.00% cellulase RS, 1.20% macerozyme R-10, 1.50% viscozyme, and 0.50 mol L^−1^ mannitol, resulting in a protoplast yield of 8.7 × 10^5^/g FW [15]. Protoplasts were prepared using *Camellia oleifera* leaves and subjected to a 10 h digestion with 1.50% cellulase R-10, 0.50% macerozyme R-10, and 0.25% snailase, resulting in a maximum yield of 3.500 × 10^7^/g FW [16]. However, the digestion time used for protoplast preparation of *C. oleifera* petals was reduced to 8 h, and the digestion solution contained 3.00% cellulase R-10, 1.00% macerozyme R-10, and 0.50 mol L^−1^ mannitol, resulting in a maximum yield of 1.42 × 10^6^/100 mg [10]. Furthermore, the yield of protoplast preparation in *Zea mays* root was significantly increased by incorporating a buffer pretreatment step [17].

RSM (Response Surface Methodology) and ANN (Artificial Neural Network) models are commonly employed for data analysis and prediction in optimization experiments across various domains, such as food [18, 19] and materials [20, 21]. The RSM model utilizes a second-order polynomial model:

$${{\text{g}}}^{0}\left({\text{x}}\right)={\text{a}}+\sum {b}_{i}{x}_{i}+\sum {c}_{i}{{x}_{i}}^{2} i=\mathrm{1,2},3,\dots n (\mathrm{Number of basic variables})$$

to study the relationship between one or more response variables and a number of independent variables, allowing for intuitive analysis of the influence of experimental factors on the results [22, 23]. Central composite, Doehlert and Box-Behnken are three main response surface design methods [24, 25] where Box-Behnken design is based on the multivariate optimization technique of three-level incomplete factorial design, which, due to its absence of axial points, helps to reduce the cost of the experiment and the number of experimental runs number of times, allowing experiments to be done more efficiently and economically [26, 27]. The ANN model is a machine learning technique that utilizes biological information to construct mathematical models for predicting the output of new datasets. Unlike RSM model, the ANN model does not require special experimental requirements or additional workload for its application [28]. Additionally, the ANN model often provides optimality-seeking results that are more aligned with experimental needs [29, 30] One of the most commonly used ANN models is the Back-Propagation Neural Network (BPNN), which adjusts the thresholds and weights in the opposite direction of the conventional method during the learning process [31, 32] The simultaneous use of RSM and ANN models can complement each other's strengths to better analyze and predict data [33, 34].

*Salsola laricifolia* belongs to the family Amaranthaceae [35]. The genus *Salsola* encompasses a diverse range of photosynthetic evolutionary types [36–38] *S. laricifolia* is classified as a type I C_3_-C_4_ intermediate plant [39]. Studying the physiological and biochemical properties of this plant type, as well as its gene functions, is crucial for understanding the evolution of the C_4_ photosynthetic pathway and the mechanism of photorespiration reduction [40–42]. Xi et al. [43] explored the preparation conditions of protoplasts of *S. laricifolia* using the orthogonal method, and have preliminarily determined the influencing factors and experiment ranges. Although obtaining a maximum yield of protoplasts of 1.210 × 10^6^/100 mg and viability of 85.00%, but the results was not stable, and could not satisfy the experimental needs. Therefore, in this study we employed the Box-Behnken experimental design and RSM-ANN model to optimize the preparation system of *S. laricifolia* protoplasts. Additionally, reference genes were chosen and the subcellular localization of the NADP-malic enzyme gene 2 (*SaNADP-ME2*) was determined for the prepared protoplasts. These steps ensured the continuation of gene research.

## Materials and methods

### Experimental materials and growth conditions

*S. laricifolia* seeds were collected from Bole, Xinjiang, China, in October 2022 and stored in a refrigerator at 4 °C. The seeds were soaked in sterile water for 1.5 h, followed by washing with a sodium hypochlorite solution (sterile water: sodium hypochlorite = 7: 3) for 7 min. During the washing process, the seeds were shaken well and rinsed 10–15 times with sterile water. After that, the seeds were treated with thiram (1 g of thiram dissolved in 20 mL of ddH_2_O) for 30 s. Subsequently, the seeds were placed on a culture medium (1/2 MS + 15 g L^−1^ sucrose + 8 g L^−1^ agar) and incubated in an artificial climate chamber. The chamber was maintained at a temperature of 25  C for 14 h during the daytime and 18  C for 10 h during the nighttime. The light intensity was set at 6000 Lux. After 3 days of germination, healthy and uniform size of seedlings were selected and transplanted into plastic pots with dimensions of 8.50 cm (height) × 10.00 cm (inner diameter) for hydroponic cultivation. The culture solution used was Hoagland's nutrient solution and changed every 3 days. The protoplast preparation experiment was conducted after 25 days.

### Reagent preparation

The reagents including mannitol, cellulase R-10, macerozyme R-10 (Solarbio, Beijing, China), MES, BSA (Sangon Biotech, Shanghai, China) were used. The formulations of pretreatment solution, enzyme digestion solution, W5 solution, WI solution, MMG solution and PEG solution were formulated as shown in Additional file 1: Table S1, and all solutions were sterilized by 0.20 µm filter. The pretreatment and enzymatic digestion solutions were prepared for use now, and when prepared, MES and mannitol were added first, and then mixed in a 55  C water bath for 10 min, and then BSA, CaCl_2_ and KCl were added sequentially after cooling down to room temperature; At last, cellulase and macerozyme were added. The PEG solution was required to be prepared for use now, and the W1 and MMG solutions could be stored in the refrigerator at 4  C for 1 week after preparation.

### Experimental methods

#### Isolation and purification of protoplasts

The steps of protoplast preparation were referred to Wang et al. [44] and Xi et al. [43] with some modifications. Using 100 mg leaves were longitudinally dissected from the middle using a sterile scalpel in an ultra-clean bench, then cut into 2–3 mm segments and placed in a pretreatment solution for 30 min. Subsequently, the segments were transferred to a 5.00 mL centrifuge tube containing 1.00 mL of enzyme digestion solution (previously heated in a 50  C water bath for 1 min). After 4 h of enzyme digestion at 26  C for 40 r min^−1^ avoiding light, the enzyme was gently inverted several times to fully release the protoplasts and placed on ice for spare time.

The W5 solution was pre-wetted on a 200 mesh sieve, after which the digest was filtered. The enzyme reaction was terminated by adding an equal volume of W5 solution to the enzyme filtrate (the pipette gun tip was cut with a 0.50 cm tip and the incision sharp filaments were removed over a flame), centrifuge the supernatant at 300 rpm min^−1^ for 2 min at 4  C and discard it, add 1 mL of W5 solution into the precipitate to resuspend the protoplasts at the bottom, and then discard the supernatant after 10 min of static time on the ice; Add 2.00 mL of W5 solution to resuspend the bottom protoplasts, leave on ice for 40 min, discard the supernatant, add 200 μL of W5 to obtain the suspension of *S. laricifolia* protoplasts, and put it in the refrigerator at 4  C for spare. Protoplast yield was calculated using hemocytometer counting method. Evans blue staining solution (0.25%, W/V) was used to dip the protoplast suspension (ratio 2: 5) for 5 min to calculate protoplast viability.

#### Box-Behnken experimental design

A Box-Behnken design of experiments using Design-Expert V8.0.6.1 software was used with protoplast yield × 10^6^/100 mg (Y_1_) and viability % (Y_2_) as response values, cellulase R-10 concentration % (W/V) as A, macerozyme R-10 concentration % (W/V) as B and mannitol concentration (mol L^−1^) as C. The factors in this experiment were designed as three levels, A (1.00, 2.00, 3.00), B (0.50, 0.75, 1.00), and C (0.50, 0.60, 0.70). The number of viable cells was calculated by multiplying the protoplast yield with the viability results obtained.

#### Comparison of RSM-ANN model

The results obtained from Box-Behnken design were analyzed by RSM and ANN models using Design Expert and Matlab R2023a, respectively, to evaluate the effect of model fitting and obtain the optimal values. The results of regression equations Y_1_ and Y_2_ obtained from the RSM model were plotted on the three-dimensional response surface plots using Origin 2021. By examining the interaction density and steepness of the curves in the plots, the magnitude of the effect of experimental factors on the response values was assessed. Typically, a steeper response surface curve indicates a greater impact of the factor interaction on the response value, while a less steep curve suggests a smaller effect [45–47]. The ANN model was analyzed using BPNN. It comprised an input layer of three neurons, an output layer of one neuron, and a variable number of hidden neurons layer, and was trained using different learning algorithms to select the set with the best fitted data, and the results of the experiments were used 70.00% for training, 15.00% for validation, and 15.00% for testing. The experimental optimums were solved by the simulation model of the BPNN using the GA in Matlab [48, 49].

After obtaining the models, their predictions were compared with the actual results, and several metrics were calculated to evaluate the goodness of fit of each model. These metrics included the correlation coefficient (R), the coefficient of determination (R^2^), the root mean square error (RMSE), and the mean absolute percentage error (MAPE). A higher value of R and R^2^ indicates a stronger correlation and better fitting ability of the model [50, 51]. Smaller values of RMSE and MAPE indicate a lower deviation and relative error between the predicted and actual values, indicating the higher accuracy in the model’s predictions [33, 52].

#### RNA extraction and gene expression analysis

After enzymatic digestion of protoplasts, the protoplasts were centrifuged at 300 rpm min^−1^ for 2 min at 4  C. RNA was extracted immediately after removing the supernatant using the MiniBEST Plant RNA Extraction Kit (Takara, Japan). The entire extraction process was performed on ice. Three sets of replicates were performed for each experiment, and each set of replicates included three technical replicates. The quality of the total RNA was assessed using the NanoDrop 2000 Ultra-Micro Spectrophotometer (Thermo, America). Subsequently, the total RNA was reverse transcribed into cDNA using the PrimeScript^™^ RT reagent kit with gDNAEraser kit (Takara, Japan). The resulting cDNA products were either used directly for quantitative Real-time Polymerase Chain Reaction (qPCR) or stored at – 20  C.

Referring to the study of Wen and Zhang [53], three housekeeping genes (*18S rRNA*, *β-actin* and *EF1-α*) were selected from *S. laricifolia*, and the comparison in expression of these genes between protoplasts and untreated leaves were analyzed by using qPCR. The qPCR primers for each housekeeping gene were in Additional file 1: Table S2, and the reaction systems were provided in Additional file materials. The relative expression of each housekeeping gene was calculated using the formula Q = E^−ΔΔCt^, where E represents the gene amplification efficiency, typically assumed to be two (100.00% efficiency). ΔCt is calculated as Ct (min)—Ct (sample), where Ct (min) is the lowest Ct value among all samples, and Ct (sample) is the Ct value of each sample [54, 55]. The data were subjected to two-sample anova and plotted using Origin.

#### Exogenous gene transformation

The *pBI121-SaNADP-ME2* and *pBI121-GFP* null-loaded *E. coli* strains were obtained from our laboratory and contained kanamycin resistance genes and *GFP* reporter gene. Refer to Ren et al. [56] for the procedure of protoplast transformation and improve it. Protoplasts were used as receptors and were first resuspended in 1 mL of MMG solution, then incubated on ice for 30 min and centrifuged at 300 rpm min^−1^ for 2 min to remove the supernatant. 600 μL of protoplast solution was pipetted into a 5 mL centrifuge tube. 60 μL of *pBI121-SaNADP-ME2-GFP* plasmid vector was added to the bottom of the tube and the tube was gently flicked to mix the contents. The tube was then inverted several times to mix the 660 μL of PEG solution. The mixture was incubated in a dark environment at room temperature for 30 min, completing the transformation process. The transformation reaction was stopped by adding a twofold volume of W5 solution. After centrifugation at 300 rpm min^−1^ for 2 min, the supernatant was discarded. The protoplasts were then resuspended in 3 mL of WI solution and placed in cell culture chambers (Labselect, Beijing, China). They were incubated in the dark at room temperature for 16–24 h. A suitable amount of the transformed protoplasts was used for further analysis. Protoplasts transformed with *pBI121-GFP* empty load were used as a control. The images were captured using a laser confocal scanning microscope (Zeiss LSM 800, Jena, Germany) and processed with Zen 2012 software. GFP and chlorophyll were excited using 488 nm and 633 nm laser lines, respectively, to observe GFP expression.

## Results

### Analysis of protoplasmic preparation results

Seventeen groups of experiments were conducted using the Box-Behnken design, and the results obtained were presented in Table 1. Among the 17 groups, group 12 exhibited the highest yield with a value of 1.566 × 10^6^/100 mg. Group 5 showed the highest viability at 90.81%, while group 12 had the maximum number of viable cells, reaching 1.367 × 10^6^/100 mg.

**Table 1:** Experimental results of three-factor, two-response-value Box-Behnken design test based on the preparation of protoplasts of *Salsola laricifolia*

Group	Cellulase R-10 content (%)	macerozyme R-10 content (%)	Mannitol concentration (mol·L^−1^)	Protoplast yield (10^6^/100 mg)	Protoplast viability (%)	Number of viable cells (yield*vitality)(10^6^/100 mg)
1	1.00	0.50	0.60	0.575	85.78	0.493
2	3.00	0.50	0.60	0.256	85.58	0.219
3	1.00	1.00	0.60	0.683	88.02	0.601
4	3.00	1.00	0.60	0.800	78.69	0.630
5	1.00	0.75	0.50	0.563	90.81	0.511
6	3.00	0.75	0.50	0.383	84.65	0.324
7	1.00	0.75	0.70	0.583	87.62	0.511
8	3.00	0.75	0.70	0.615	84.27	0.518
9	2.00	0.50	0.50	1.125	90.10	1.013
10	2.00	1.00	0.50	1.460	90.62	1.323
11	2.00	0.50	0.70	1.320	89.23	1.178
12	2.00	1.00	0.70	1.566	87.27	1.367
13	2.00	0.75	0.60	0.955	89.63	0.856
14	2.00	0.75	0.60	0.945	89.42	0.845
15	2.00	0.75	0.60	1.035	88.25	0.913
16	2.00	0.75	0.60	1.090	88.57	0.965
17	2.00	0.75	0.60	1.010	87.21	0.881

### RSM results analysis

#### RSM regression model analysis

The test results were analyzed using RSM to establish the response values Y_1_ and Y_2_ based on the experimental factors A, B, and C. Quadratic multinomial regression equations was obtained. The model evaluation indexes were in Table 2, and the detailed results of the model were provided in Additional file 1: Tables S3, S4.

$${\text{Y}}_{{1}} = 6.119 + 1.815{\text{A}} - {4}{\text{.626B}} - {18}{\text{.608C + 0}}{\text{.436AB + 0}}{\text{.580AC}} - {0}{\text{.887BC}} - {0}{\text{.633A}}^{2} + 3.269{\text{B}}^{2} + 15.650{\text{C}}^{2}$$

$${\text{Y}}_{{2}} = 18.883 + 13.360{\text{A + 49}}{\text{.779B}} - {186}{\text{.192C}} - {9}{\text{.063AB + 7}}{\text{.010AC}} - {24}{\text{.790BC}} - {3}{\text{.289A}}^{2} - 13.194{\text{B}}^{2} + 150.865{\text{C}}^{2}$$

**Table 2:** Response surface model evaluation indexes

Evaluation metrics	Protoplast yield model	Protoplast viability model
F-value	105.270	21.080
P-value	< 0.0001	0.0003
R^2^	0.993	0.964
R^2^ of experiment value	0.983	0.919
R^2^ of predicted value	0.976	0.815
Coefficient of variation (C.V. %)	5.46	0.98
signal-to-noise ratio	35.072	18.012

The yield model (Y_1_) had an F-value of 105.270, indicating highly significant level differences (*p* < 0.0001). The R^2^ value was 0.993, and the signal-to-noise ratio was 35.072, which was greater than 10, indicating that the model was sufficiently accurate and not easily affected by external disturbances. Additionally, the data had an acceptable level of variability, as evidenced by the range of 4% to 10% for the coefficient of variation (C.V. %). The viability model (Y_2_) had an F-value of 21.080 (*p* = 0.0003), which was less than 0.010, indicating a significant model variance. The R^2^ value was 0.964, and the signal-to-noise ratio was 18.012, indicating a high level of accuracy and resistance to external disturbances. Additionally, the C.V.% value was less than 4.00, suggesting a low degree of data variability in the model.

#### RSM model three-dimensional result graph analysis

**Fig. 1:**
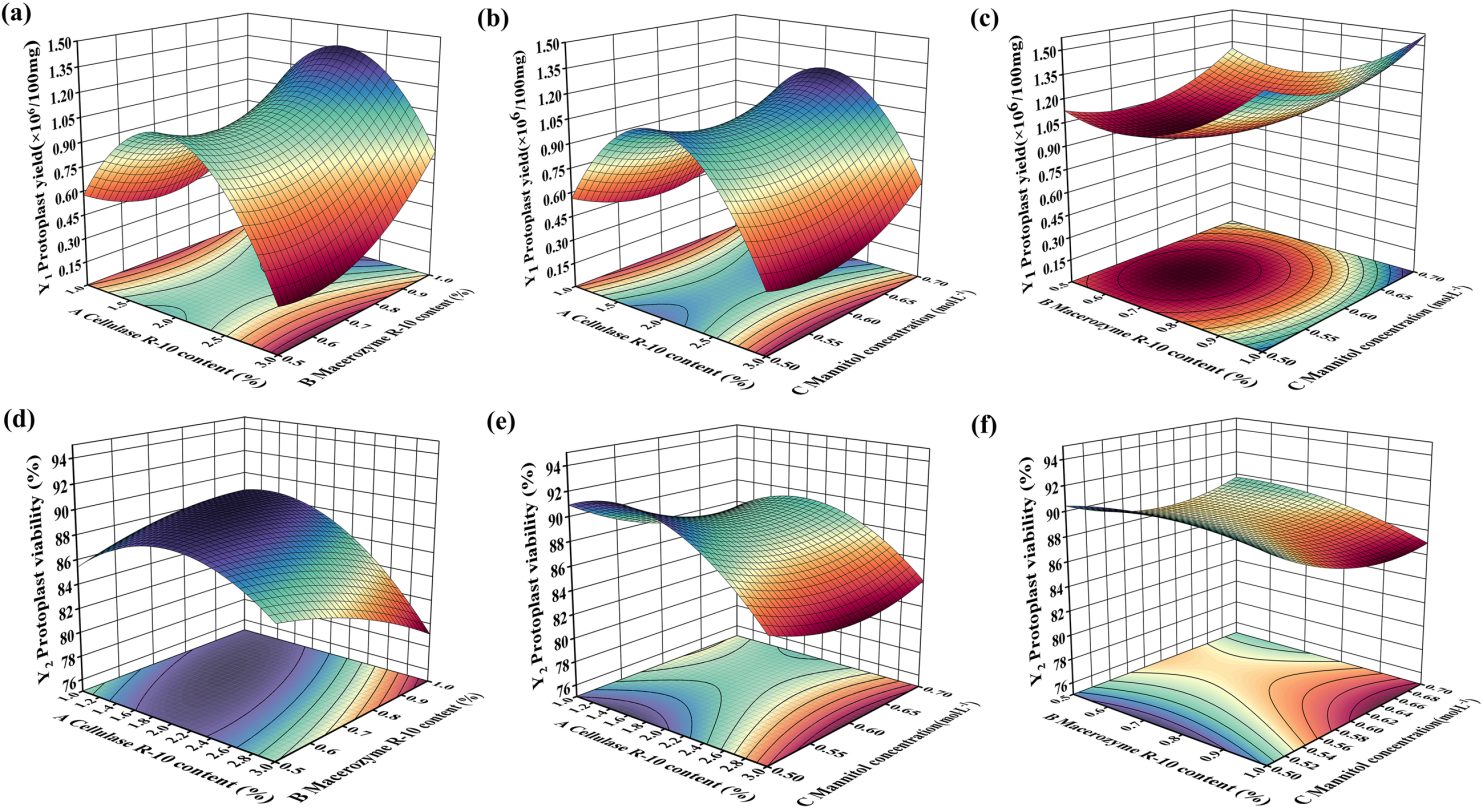
Three-dimensional response surface plots based on the RSM model resolving the interaction of various factors of the experiment on the results of protoplast preparation of *Salsola laricifolia*. The plots labeled **a**, **b**, and **c** represent the protoplast yield (Y_1_) and show how it is influenced by the interaction between two experimental factors, Similarly, the plots labeled **d**, **e**, and **f** represent protoplast viability (Y_2_) and how it is influenced by the interaction between two experimental factors. X-axis, Y-axis are the experimental factors including cellulase R-10 content, macerozyme R-10 content, and mannitol concentration, denoted by A, B, and C, respectively

From Fig. 1a, b and c, it can be observed that the interaction effects of cellulase R-10 content (A) and macerozyme R-10 content (B) were the most significant. The influence of A on protoplast yield (Y_1_) was greater than that of B, which was manifested by the fact that the surface intersecting AB was the steepest, and the contours intersecting with the A-axis were more densely packed than those of the B-axis (Fig. 1a), the interaction between A and mannitol concentration (C) had more significant effect on Y_1_. A had a greater effect on Y_1_ than C (Fig. 1b), where the surface was smoother and the contours intersecting the A-axis were denser than those of the C-axis. The interaction between B and mannitol concentration (C) had a significant effect, and B had a greater effect on Y_1_ than C (Fig. 1c), where the surface was smoothest and the contours intersecting the B-axis were denser than those of the C-axis. Therefore, it can be concluded that the effects of the experimental factors A, B, and C on Y_1_ following the order of A > B > C. Additionally, the effect of the interaction between these factors on Y1 followed the order of AB > AC > BC.

Similarly, as shown in Figs. 1d, 1e, and f, the experimental factors on the protoplast preparation viability (Y_2_) resulted in the order of the influence of B > A > C, the interaction between the factors on the degree of influence of Y_2_ in the order of AB > BC > AC.

### ANN result analysis

**Fig. 2:**
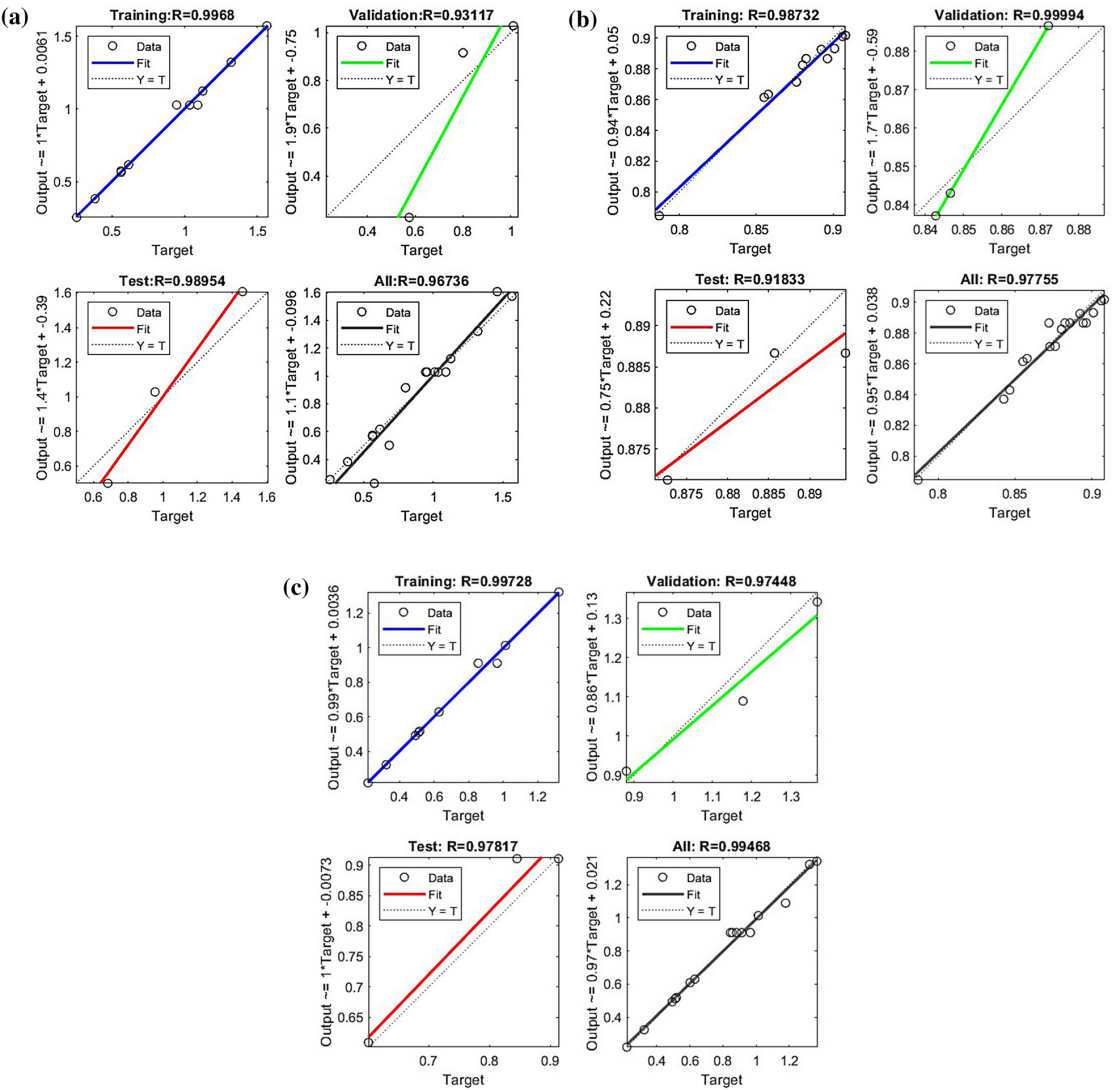
Artificial neural network (ANN) model simulation effect. It displays the training, verification, test and all data fitting for each model. The fitting effect is represented by correlation coefficient (R). **a** represents the yield model, **b** represents the viability model, and **c** represents the viable cell model

The mean square values, error values, and simulation effects of the ANN model were shown in Fig. 2. The yield model was optimized using the Levenberg–Marquardt algorithm with 18 hidden layers. The vitality model was optimized using the scaled conjugate gradient method with 17 hidden layers. The viable cell model was optimized using the Levenberg–Marquardt algorithm with 14 hidden layers.

**Fig. 3:**
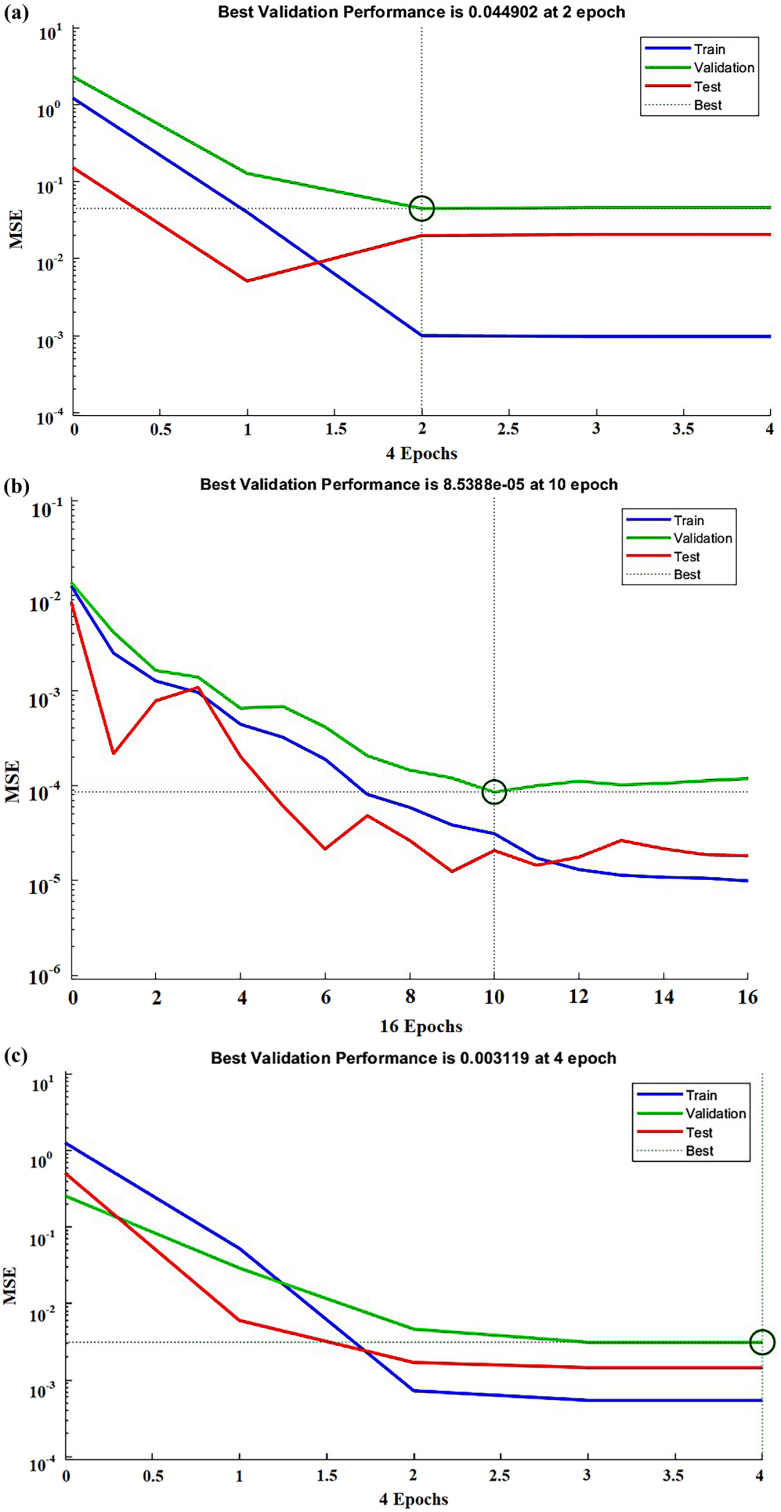
Artificial neural network (ANN) model mean square error (MSE) effect plot. The top shows the number of ANN model epochs when the minimum MSE is obtained for validation data. **a** represents the yield model, **b** represents the vigor model, and **c** represents the viable cell model

The correlation coefficient of the yield model obtained after training the ANN model was 0.96736. The end-of-training condition was met after 5 iterations, with a mean square error (MSE) of 0.045 (Figs. 2, 3a). The correlation coefficient of the ANN-viability model was 0.977, and the MSE of the ANN-viability model was 8.5388 e^−05^ after 10 iterations, satisfying the end-of-training condition (Figs. 2, 3b). The correlation coefficient of the ANN-vital cell model was 0.995, and its mean square error was 0.003 after four iterations, also satisfying the end-of-training condition (Figs. 2, 3c).

### Comparison between RSM and ANN model fitting and prediction performance

#### Fitting effect of RSM, ANN

**Fig. 4:**
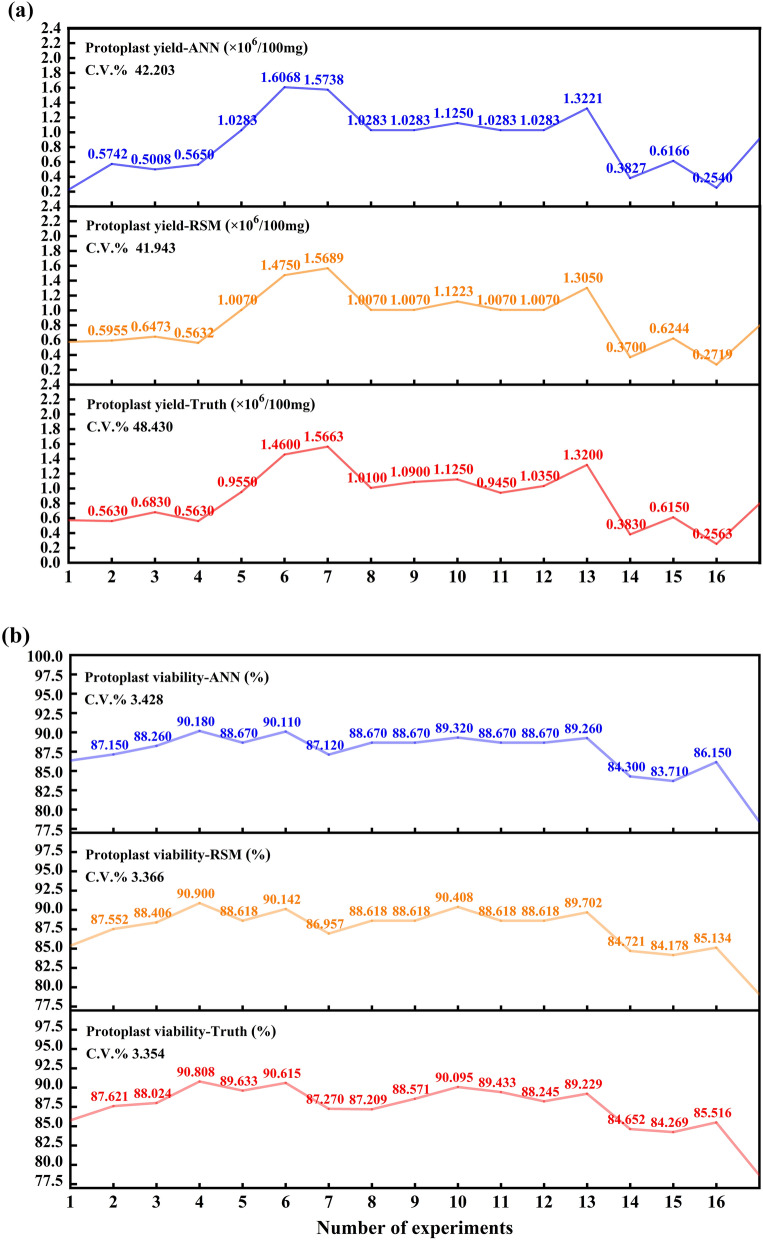
Comparison of experimental values with predicted values of response surface methodology (RSM), artificial neural network (ANN) models. **a** for protoplast yield, **b** for protoplast viability

According to Fig. 4, the RSM yield and viability model had a better fit compared to the ANN yield and viability model. The R and R^2^ values of the RSM model were 0.996, 0.992, and 0.982, 0.964 respectively, which were closer to 1 than the values of the ANN model. Additionally, the RSM model had smaller RMSE and MAPE values compared to the ANN model. However, the ANN model showed improved accuracy in predicting the number of live cells, with its R and R^2^ values being second only to the best-fitting RSM yield model (Fig. 4, Table 3).

**Table 3:** Evaluation of response surface methodology (RSM), artificial neural network (ANN) model based on statistical indexes

Statistical indexes	RSM-yield	RSM-viability	ANN-yield	ANN-viability	ANN-number of viable cells
R	0.996	0.982	0.967	0.977	0.994
R^2^	0.992	0.964	0.935	0.955	0.989
RMSE	0.034	0.005	0.110	0.006	0.035
MAPE/%	2.825	0.478	8.274	0.595	2.009

*R* correlation coefficient, *R*^*2*^ coefficient of determination, *RMSE* root mean square error, *MAPE* mean absolute percentage error

When analyzing only yield and viability, protoplast yield fluctuated greatly by changes in experimental conditions, while viability fluctuated less. The coefficients of variation (C.V.%) of the actual and predicted values of protoplast yield were 42.203, 41.943 and 48.438, which were much larger than those of the actual and predicted values of viability, which were 3.428, 3.366, and 3.354 (Fig. 4). Consequently, the RMSE and MAPE of the predicted values for yield were higher than those for viability predictions in Table 3, indicating a greater bias in yield prediction.

#### RSM, ANN model optimization and verification

The RSM model was used to solve for the optimal values under the conditions of simultaneous consideration of the yield and viability. The results revealed that under the conditions of a cellulase R-10 content of 1.86%, macerozyme R-10 content of 1.00% and mannitol concentration of 0.50 mol L^−1^, the theoretical values for protoplast yield were 1.464 × 10^6^/100 mg and viability was 90.81% (Table 4). GA was utilized for optimization of ANN-yield model, ANN-viability model and ANN-viable cell number model respectively. ANN-yield model predicted a theoretical maximum protoplast yield of 1.625 × 10^6^/100 mg. This prediction was based on the following experimental conditions: 1.98% cellulase R-10 content, 1.00% macerozyme R-10 content, and 0.50 mol L^−1^ mannitol concentration. Similarly, the viability model predicted a theoretical maximum viability of 96.29 based on experimental conditions including 1.65% cellulase R-10 content, 0.98% macerozyme R-10 content, and 0.53 mol L^−1^ mannitol concentration. Additionally, the ANN viable cell number model predicted a theoretical maximum viable cell number of 1.342 × 10^6^/100 mg, which was based on 2.00% cellulase R-10 content, 1.00% macerozyme R-10 content, and 0.70 mol L^−1^ mannitol concentration (Table 4). To validate these predictions, the predicted values, experimentally validated values, and relative errors of the results were calculated under the respective optimal conditions of the RSM and ANN models, and Table 4 was obtained.

**Table 4:** The response surface methodology (RSM) yield-vigor model, the artificial neural network(ANN) yield model, the vigor model, and the viable cell number model predictions were validated, and the results were considered credible with a relative error of less than 5%

Model	Cellulase R-10 content (%)	Macerozyme R-10 content (%)	Mannitol concentration(mol L^−1^)	Protoplast yield(× 10^6^/100 mg)		Protoplast viability (%)		Number of viable cells(× 10^6^/100 mg)		Relative error (%)		
				Predicted value	Experiment value	Predicted value	Experiment value	Predicted value	Experiment value	Protoplast yield	Protoplast viability	Number of viable cells
RSM yield-viability	1.86	1.00	0.50	1.464	1.470	90.81	89.73	1.329	1.319	0.41	1.20	0.76
ANN-yield	1.98	1.00	0.50	1.625	1.550	90.16	90.65	1.337	1.405	4.84	0.54	4.83
ANN-viability	1.00	0.75	0.50	0.565	0.563	90.18	90.81	0.510	0.508	0.36	0.69	0.39
ANN-number of viable cells	2.00	1.00	0.70	1.574	1.566	87.12	87.27	1.342	1.367	0.51	0.17	1.83

**Fig. 5:**
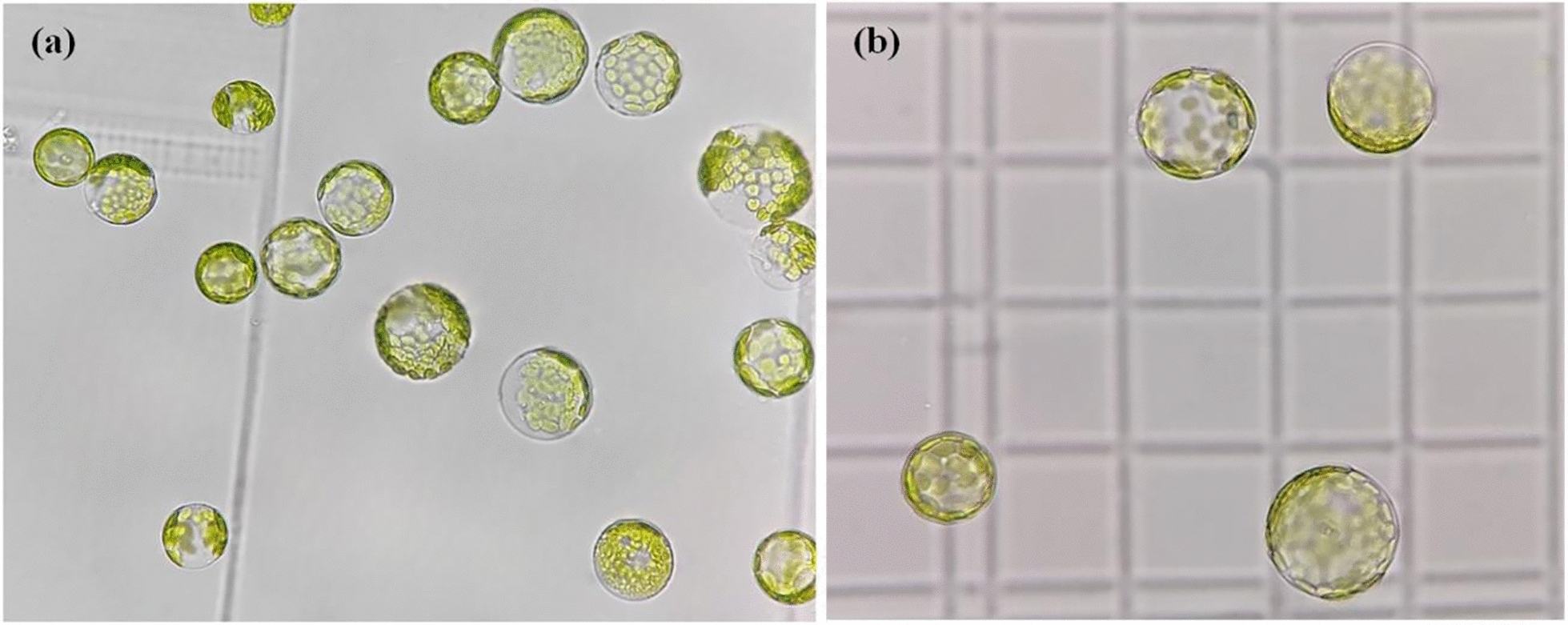
Results of the preparation of protoplasts of *Salsola laricifolia* under the optimal preparation conditions. To enhance visualization of protoplasts, **a** and **b** were captured using blood counting plates (25 × 16) as a background under a microscope (10 × 40). Additionally, the protoplast concentrations in **b** were diluted by a factor of two

From Table 4, the relative errors between the prediction results and the actual results using RSM and ANN models were less than 5.00%, indicating a high degree of confidence in the prediction results of both models [57]. Considering the protoplast yield, viability and viable cell number, the ANN yield model predicted the optimal results, and the validation resulted in a protoplast yield of 1.550 × 10^6^/100 mg, the viability of 90.65% and a viable cell number of 1.405 × 10^6^/100 mg, which corresponded to the preparation conditions of 1.98% cellulase R-10 content, 1.00% macerozyme R-10 content and 0.50 mol L^−1^ mannitol concentration. When compared to the RSM model prediction validation results (1.470 × 10^6^/100 mg, 89.73%, 1.319 × 10^6^/100 mg), the indicators showed improvements of 5.44%, 1.03%, and 6.52% respectively. Figure 5 demonstrates the results of the protoplast preparation under these conditions.

### Reference gene stability analysis

**Fig. 6:**
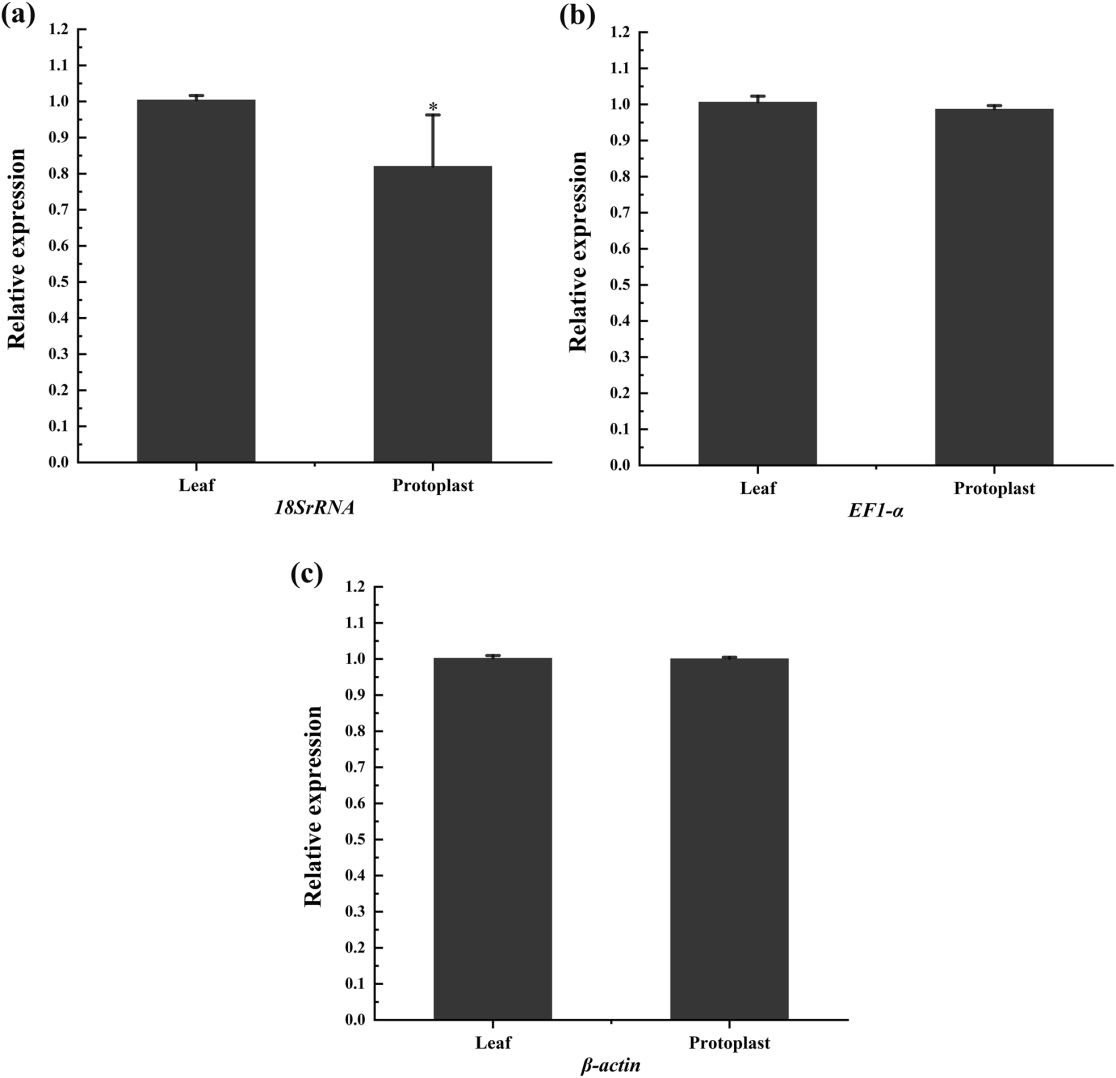
Relative expression of the three housekeeping genes (**a**, **b**, **c** represent *18SRNA, EF1-α,* and *β-actin*, respectively) in the protoplasts and leaves of *Salsola laricifolia*. _*_ represents significant differences (*p* < 0.05)

The A_260_/A_280_ and A_260_/A_230_ ratios of RNA in both protoplasts and untreated leaves of *S. laricifolia* were approximately 2.00, which satisfied the requirements for subsequent experiments. The relative expression of the three housekeeping genes in both protoplasts and leaves was shown in Fig. 6. There was no significant difference in the expression of *β-actin* and *EF1-α* pre-and post-treatments. However, there was a significant difference in the expression of *18SrRNA* (*p* < 0.05), indicating that it could not be used as an reference gene in the protoplast experiment.

### Results of protoplast transformation

**Fig. 7:**
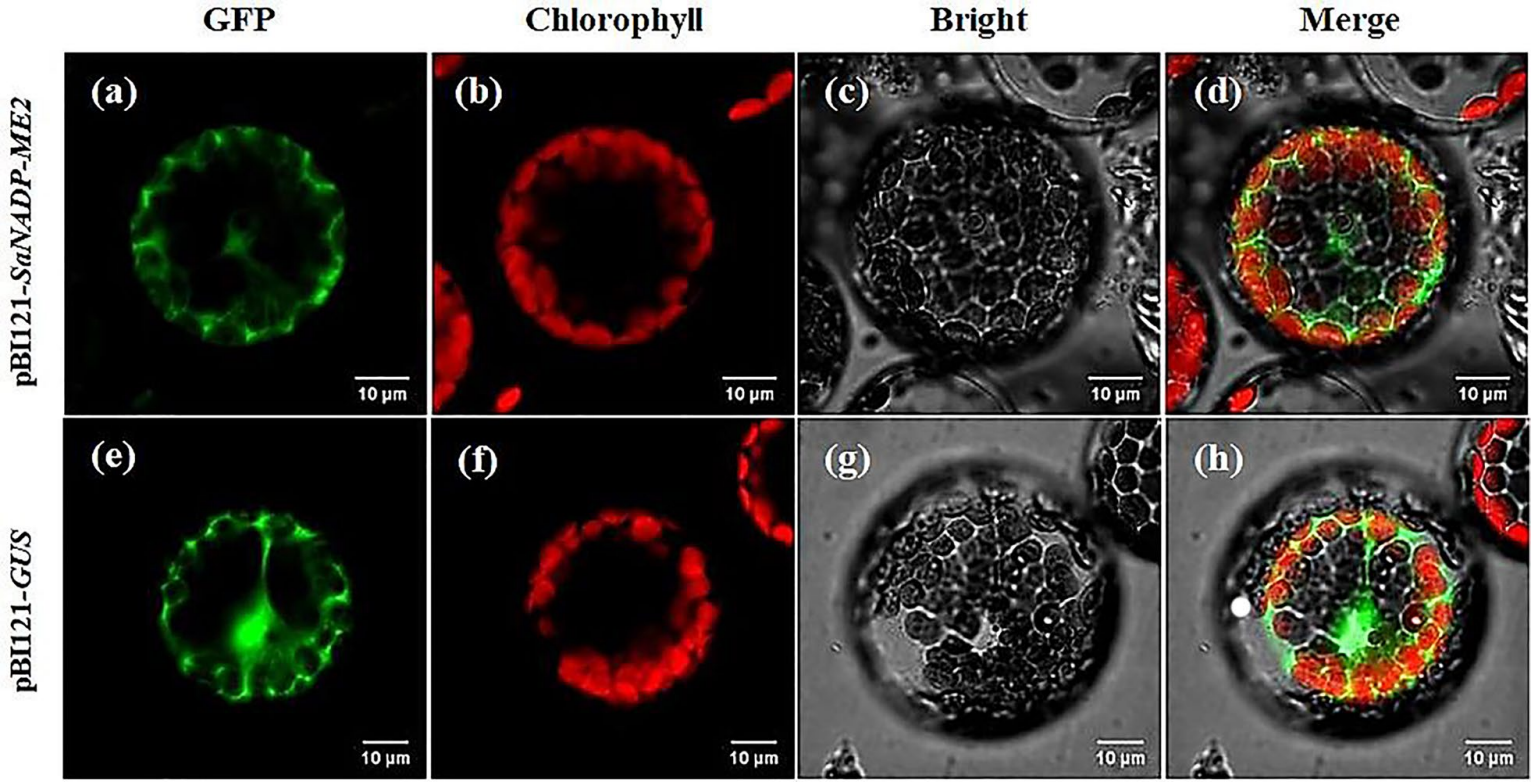
Transformation results of protoplasts under laser confocal scanning microscope, green and red fluorescence indicate the localization of GFP protein and chlorophyll, respectively. **a**, **b**, **c**, **d**: sequentially, *pBI121-SaNADP-ME2* plasmid transformed protoplasts in GFP fluorescence, chloroplast autofluorescence, white light and three images superimposed; **e**, **f**, **g**, **h**: sequentially, *pBI121-GFP* empty plasmid transformed protoplasts in GFP fluorescence, chloroplast autofluorescence, white light and three images superimposed

The results of the transformation of *S. laricifolia* protoplasts using the *pBI121-SaNADP-ME2* plasmid vector were shown in Fig. 7, where fluorescence signals of GFP and chlorophyll were detected respectively at 500–530 nm and 650–750 nm after excitation at 488 nm and 633 nm. The protoplasts transformed with *pBI121-SaNADP-ME2* plasmid vector could observe green fluorescence of GFP protein in chloroplasts (Figs. 7a, b, c, d), while protoplasts imported with empty plasmid only emitted green fluorescence in cytoplasm, with no obvious fluorescence in chloroplasts (Figs. 7e, f, g, h). The protoplast cells that were successfully transformed appeared transparent and had a regular shape without any breakage.

## Discussion

In this study, we utilized RSM and ANN models to predict the best preparation conditions for *S. laricifolia* protoplasts. Our findings revealed that the Box-Behnken design test provided the necessary data for our analysis. The RSM model demonstrated a better fit compared to the ANN model (Table 3). However, the final protoplast preparation conditions were determined using the ANN-yield model (Table 4). Because the amount of protoplast yield was fluctuated more when changing experimental conditions compared to protoplast viability, leading to the fact that protoplast yield tends to be more determinative of the number of viable cells (Table 3, Fig. 4). Although the ANN-yield model produced a viability value of 90.65%, slightly lower than the predicted value of the ANN-viability model (90.81%), the final number of viable cells obtained was 1.405 × 10^6^/100 mg, which was the highest value (Table 4). As a result, the preparation conditions predicted by the ANN-viability model were chosen as the final conditions. Compared to the previous experimental results of *S. laricifolia* protoplasts [41], the protoplast yield increased by 28.10%, the protoplast viability increased by 6.65%, and the number of viable cells increased by 36.61%. Furthermore, when compared to the best results before the ANN treatment (Table 1), there was an additional increase of 3.87% in the protoplast viability and 2.78% in the number of viable cells.

After modeling the ANN model using the number of viable cells, the fitting effect of the ANN model was significantly improved, with R, and R^2^ were next only to the best-fitting RSM-yield model, and the relative error between the experimental validation results and the predicted results was also less than 5.00% (Tables 3, 4). Therefore, it is recommended to consider the number of living cells when using the ANN model to optimize the results of protoplast preparation. This consideration, along with the analysis of protoplast yield and viability, can help obtain predicted results that align more closely with experimental expectations. With the further application of deep learning in cell research, studies have been conducted to identify dead/surviving cells directly using convolutional neural networks [58], which makes it possible to count the number of living cells more easily. Additionally, the fitting ability of RSM and ANN models varies depending on the predicted objects [33, 52, 59–61]. Therefore, when different materials are used and steps are taken to prepare protoplasts, they need to be evaluated in detail with specific evaluation indexes and experimental data.

In this study, the expression differences of three housekeeping genes (*18sRNA, EF1-α,* and *β-actin*) before and after the preparation of pine leaf pigweed protoplasts. were shown in Fig. 6. We optimized the protoplast preparation system and identified suitable internal reference genes to avoid any potential alteration of their expression in the pigweed protoplasts, which could affect the results of subsequent experiments. Housekeeping genes are essential for maintaining minimum cellular functions and are generally considered to be stably expressed [62–65] However, their expression may be altered to varying degrees under different adverse conditions. Unlike *β-actin* and *EF1-α*, the expression of *18sRNA* selected in this experiment were found to be unstable and unsuitable for use as endogenous genes after the preparation process of *S. laricifolia* protoplasts (Fig. 5), this aligns with the findings of a previous study that investigated the suitability of these genes as endogenous genes in *S. laricifolia* leaves under drought stress [53].

## Conclusions

In this experiment, Box-Behnken design method with RSM-ANN model was used to optimize the preparation conditions of protoplasts of *S. laricifolia*, these methods are not commonly used in previously similar studies. To validate the methodology, a series of experiments were conducted, including transient gene conversion and internal reference gene analysis. After considering the protoplast yield, viability and number of viable cells, the ANN yield model predicted the best results, and the experimental validation protoplast yield of 1.550 × 10^6^/100 mg, viability of 90.65%, and a number of viable cells of 1.405 × 10^6^/100 mg. Corresponding preparation conditions were 1.98% cellulase R-10, 1.00% macerozyme R-10, 0.50 mol L^−1^ mannitol. Furthermore, *β-actin* and *EF1-α* were identified as internal reference genes in protoplast experiment. The experiments also demonstrated that, in addition to protoplast yield and viability, the number of viable cells (yield × viability) can serve as an evaluation index for predicting protoplast experiments using RSM and ANN. This index not only considers the viability and yield of protoplasts simultaneously but also aligns better with the requirements of subsequent genetic experiments.

### Supplementary Information

**Additional file 1: Table S1.** Components of experimental reagents. **Table S2.** Primer sequences of three housekeeping genes in *Salsola laricifolia*. **Table S3.** Regression analysis of response surface yield and vitality models, *, **, and *** indicate significant differences at the *p* < 0.05, *p* < 0.01, and *p* < 0.001 levels, respectively. **Table S4.** Fit statistics of Box-Behnken design-response surface methodology. *Salsola laricifolia* housekeeping gene reaction system in the supplementary Information.

## Data Availability

Not applicable.
