# Supplementary material for: Optimization of preparation conditions for Salsola laricifolia protoplasts using response surface methodology and artificial neural network modeling
Source: Plant Methods. 2024 Apr 7;20:52. doi: 10.1186/s13007-024-01180-9 (PMC11000288; doi:10.1186/s13007-024-01180-9)
Supplement: Supplementary file 1 — Additional file 1: Table S1. Components of experimental reagents. Table S2. Primer sequences of three housekeeping genes in Salsola laricifolia. Table S3. Regression analysis of response surface yield and vitality models, *, **, and *** indicate significant differences at the p < 0.05, p < 0.01, and p < 0.001 levels, respectively. Table S4. Fit statistics of Box-Behnken design-response surface methodology. Salsola laricifolia housekeeping gene reaction system in the supplementary Information. [file 13007_2024_1180_MOESM1_ESM.docx]

Table S1: Components of experimental reagents

| Name | Ingredients |
| --- | --- |
| pretreatment solution | 2.00 M KCl 100μL,1M CaCl_2_ 100 μL,5.00% BSA（W/V）200 μL,0.80 M mannitol 7.50mL, 0.20 M MES (KOH adjusted pH to 7.5) 1.00 ml, mixed with deionized water to 10.00mL. |
| enzyme digestion solution | 1.00 M CaCl_2_ 10 μL,0.20 M MES (KOH adjusted pH to 7.5) 100μL,5.00% BSA（W/V）20μL, cellulase, macerozyme and mannitol concentration ratios are shown in Table 1 of the article,mixed with deionized water to 1.00mL. |
| W5 solution | 0.900 g·L^-1^ glucose, 9.000 g·L^-1^ NaCl, 13.875 g·L^-1^ CaCl_2_, 0.370 g·L^-1^ KCl, 0.853 g·L^-1^ MES,KOH adjusted pH to 5.8. |
| WI solution | 73.000 g·L^-1^ mannitol, 0.853 g·L^-1^ MES, 1.490 g·L^-1^ KCl, KOH adjusted pH to 5.8. |
| MMG solution | 73.000 g·L^-1^ mannitol, 1.430 g·L^-1^ MgCl_2_, 0.853 g·L^-1^ MES, KOH adjusted pH to 5.8. |
| PEG solution | 400.000 g·L^-1^ PEG_4000_, 36.500 g·L^-1^ mannitol, 11.100 g·L^-1^ CaCl_2_, KOH adjusted pH to 5.8. |

Table S2: Primer sequences of three housekeeping genes in *Salsola laricifolia*

| Gene | Primer sequence (5’→3’) | Amplification product size/bp |
| --- | --- | --- |
| 18S rRNA | F: GGGCATTCGTATTTCATAGTCA;  R: CGGCATCGTTTATGGTTGA | 159 |
| $\beta$-actin | F: TCCACGAAACAACCTACAACTC;  R: CAGCAATACCGGGGAACAT | 111 |
| EF1-$\alpha$ | F: TCAGTTTGGTGGTTATTGGACA;  R: ACCTCTTGTTCATCTCAGCAGC | 135 |

Table S3: Regression analysis of response surface yield and vitality models, *, **, and *** indicate significant differences at the P < 0.05, P < 0.01, and P < 0.001 levels, respectively.

| Source of variation | Sum of squares | Degrees of freedom | Mean square | F-value | P-value | Significance |
| --- | --- | --- | --- | --- | --- | --- |
| Yield model | 2.19 | 9 | 0.24 | 105.27 | < 0.0001 | *** |
| A- Cellulase R-10 content | 0.014 | 1 | 0.014 | 5.86 | 0.0461 | * |
| B- Macerozyme R-10content | 0.19 | 1 | 0.19 | 82.37 | < 0.0001 | *** |
| C- Mannitol concentrations | 0.036 | 1 | 0.036 | 15.41 | 0.0057 | ** |
| AB | 0.047 | 1 | 0.047 | 20.58 | 0.0027 | ** |
| AC | 0.013 | 1 | 0.013 | 5.83 | 0.0464 | * |
| BC | 1.969E^-3^ | 1 | 1.969E^-3^ | 0.85 | 0.3863 |  |
| A^2^ | 1.69 | 1 | 1.69 | 730.72 | < 0.0001 | *** |
| B^2^ | 0.18 | 1 | 0.18 | 76.19 | < 0.0001 | *** |
| C^2^ | 0.10 | 1 | 0.10 | 44.70 | 0.0003 | ** |
| Residual error | 0.016 | 7 | 2.307E^-3^ |  |  |  |
| Missing fit | 1.919E^-3^ | 3 | 6.397E^-4^ | 0.18 | 0.9049 |  |
| Pure error | 0.014 | 4 | 3.558E^-3^ |  |  |  |
| Calibration sum | 2.20 | 16 |  |  |  |  |
| Viability model | 138.47 | 9 | 15.39 | 21.08 | 0.0003 | ** |
| A- Cellulase R-10 content | 45.63 | 1 | 45.63 | 62.52 | < 0.0001 | *** |
| B- Macerozyme R-10 content | 4.53 | 1 | 4.53 | 6.21 | 0.0415 | ** |
| C- Mannitol concentrations | 7.57 | 1 | 7.57 | 10.37 | 0.0147 | * |
| AB | 20.53 | 1 | 20.53 | 28.13 | 0.0011 | ** |
| AC | 1.97 | 1 | 1.97 | 2.69 | 0.1448 |  |
| BC | 1.54 | 1 | 1.54 | 2.10 | 0.1901 |  |
| A^2^ | 45.56 | 1 | 45.56 | 62.42 | < 0.0001 | *** |
| B^2^ | 2.86 | 1 | 2.86 | 3.92 | 0.0881 |  |
| C^2^ | 9.58 | 1 | 9.58 | 13.13 | 0.0085 | ** |
| Residual error | 5.11 | 7 | 0.73 |  |  |  |
| Missing fit | 1.29 | 3 | 0.43 | 0.45 | 0.7314 |  |
| Pure error | 3.82 | 4 | 0.96 |  |  |  |
| Calibration sum | 143.58 | 16 |  |  |  |  |

Table S4: Fit statistics of Box-Behnken design- response surface methodology (RSM)

| Fit statistics | RSM-yield model | RSM-viability model |
| --- | --- | --- |
| Std.Dev. | 0.05 | 0.85 |
| C.V.% | 5.46 | 0.98 |
| R-Squared | 0.99 | 0.96 |
| Adj R-Squared | 0.98 | 0.92 |
| Pred R-Squared | 0.98 | 0.81 |
| Adeq Precisior | 35.07 | 18.01 |

Note: Std.Dev..standard deviation, C.V.%, coefficient of variation, The closer R-Squared and Adj R-Squared are to 1.00, the better the model fit is, Adj R-Squared minus Pred R-Squared less than 0.2 indicates a good model fit, Adeq Precisior greater than 10 that the model is less susceptible to outside interference.

*Salsola laricifolia* housekeeping gene reaction system:

The qPCR analysis of each housekeeping gene was carried out using the CFX96 Real-Time PCR Detection System (Bio-Rad, USA), following the instruction manual of TB Green Premix Ex Taq^TM^ II (Takara). Each sample was repeated three times, and three technical replicates were performed for each sample.

The reaction conditions consisted of a two-step pre-denaturation at 95℃ for 30 seconds, followed by denaturation at 95℃ for 5 seconds, annealing at 60℃ for 30 seconds, and 40 cycles.

Upon completion, the fluorescence signal (Ct value) of the dissolution curve was recorded.
